# Evaluating atherogenic index of plasma as a predictor for metabolic syndrome: a cross-sectional analysis from Northern Taiwan

**DOI:** 10.3389/fendo.2024.1438254

**Published:** 2025-01-13

**Authors:** Liang-Sien Chen, Yu-Rui Chen, Yi-Hsiu Lin, Hung-Keng Wu, Yan Wen Lee, Jau-Yuan Chen

**Affiliations:** ^1^ Department of Family Medicine, Chang-Gung Memorial Hospital, Taoyuan, Taiwan; ^2^ College of Medicine, Chang Gung University, Taoyuan, Taiwan; ^3^ Department of Medication Education, Chang-Gung Memorial Hospital, Taoyuan, Taiwan; ^4^ School of Public Health, Taipei Medical University, Taipei, Taiwan

**Keywords:** atherogenic index of plasma, triglycerides, high-density lipoprotein cholesterol, metabolic syndrome, women

## Abstract

**Background:**

The rising global prevalence of metabolic syndrome (MetS), characterized by a constellation of cardiovascular risk factors, underscores the urgent need to identify reliable predictive biomarkers. We hypothesize that an elevated atherogenic index of plasma (AIP) predicts MetS risk through lipid imbalance, but population-specific variations in its predictive strength remain unexplored. Our study aimed to assess AIP), a ratio of triglycerides to high-density lipoprotein cholesterol, as a predictor of MetS.

**Method:**

Between 2014 and 2018, our cross-sectional study collected and analyzed health examination data from 9,202 Northern Taiwan Medical Center employees without cardiovascular diseases, diabetes, and end-stage renal disease (ESRD). Our study classified AIP levels equally into three tertiles and evaluated their impact on MetS through a logistic regression model.

**Results:**

After adjusting for age, gender, BMI, SBP, FPG, and LDL in our models, the ORs for MetS in the second and third tertiles of the AIP were 3.81 (95% CI: 2.33 to 6.21; OR: 37.14, 95%: 23.22 to 59.39). In addition, women have a higher MetS risk associated with elevated AIP than men across all models.

**Conclusion:**

Our research identified the AIP as a significant predictive marker for the prevalence of MetS, suggesting its potential utility in clinical risk assessment and indicating the need for further research to explore its application in preventive strategies and therapeutic interventions.

## 1 Introduction

Metabolic syndrome (MetS) represents a clustering of cardiovascular risk factors that significantly elevate the risk of developing heart disease, stroke, and diabetes. These factors include increased blood pressure, high blood sugar, excess body fat around the waist, and abnormal cholesterol or triglyceride levels. The prevalence of MetS has been rising globally, concomitant with the obesity epidemic, making it a critical focus for public health initiatives (1–3). Therefore, identifying biomarkers that can predict the development of MetS is paramount for early intervention strategies.

The atherogenic index of plasma (AIP), a logarithmic calculation based on the ratio of triglycerides (TG) to high-density lipoprotein cholesterol (HDL-C), has emerged as a potent predictor of atherosclerosis and cardiovascular disease (CVD) (4–6). Recent studies suggest that the AIP could also be intricately linked to MetS, providing a simple yet effective tool for gauging metabolic health and the risk of cardiovascular complications (7–9). The relevance of the AIP as a predictive marker for MetS underscores the need for comprehensive research to further elucidate this relationship, which could lead to better predictive models for cardiovascular risk.

A 9-year longitudinal study in Taiwan highlighted AIP’s strong predictive value for MetS, hypertension, and T2DM, particularly among middle-aged individuals, while a 15-year study confirmed its role as an independent predictor of MetS in men, showing a significant linear trend with increasing tertiles. In India, AIP demonstrated the highest diagnostic accuracy (AUC 0.954) for MetS, and studies in chronic kidney disease and schizophrenia populations further emphasized its robust association with MetS risk factors. A Moroccan study linked elevated AIP, TG levels, and reduced HDL-C to increased cardiovascular risk, surpassing lipid measures alone (7–9). In a cross-sectional analysis, the Atherogenic Index of Plasma (AIP) was highlighted as a predictive marker for Metabolic Syndrome (MetS). A study of chronic kidney disease patients on hemodialysis found a strong correlation between elevated AIP and MetS prevalence, emphasizing its potential in cardiovascular risk management. In schizophrenia patients, AIP showed high diagnostic accuracy for MetS, with an AUC of 0.845 and a cutoff of 0.4. Similarly, research among Moroccan women demonstrated stronger associations of lipid ratios and AIP with cardiovascular risks than individual lipids, suggesting AIP’s vital role in identifying metabolic health risks across diverse populations (10–12). These findings underscore AIP’s critical value in early detection, risk stratification, and intervention strategies across various clinical and demographic settings.

The relationship between AIP and MetS is essential to address the rising prevalence of MetS, a key contributor to cardiovascular diseases and diabetes. AIP, a biomarker derived from TG and HDL-C, shows promise in predicting MetS risk across populations. However, the predictive strength of AIP varies, and population-specific insights are limited. Investigating AIP’s role in MetS can enhance early detection, risk stratification, and intervention strategies, bridging gaps in understanding its utility and offering a simple yet effective tool for managing cardiometabolic health across diverse clinical and demographic settings. This study aimed to explore the association between the AIP and MetS by leveraging a robust analytical approach to understand the extent of their correlation. Through a detailed analysis of the association between AIP and MetS, our research seeks to add a significant piece to the puzzle of metabolic health, with implications for clinical practices and public health policies.

## 2 Materials and methods

### 2.1 Data collection and population

**Figure 1:**
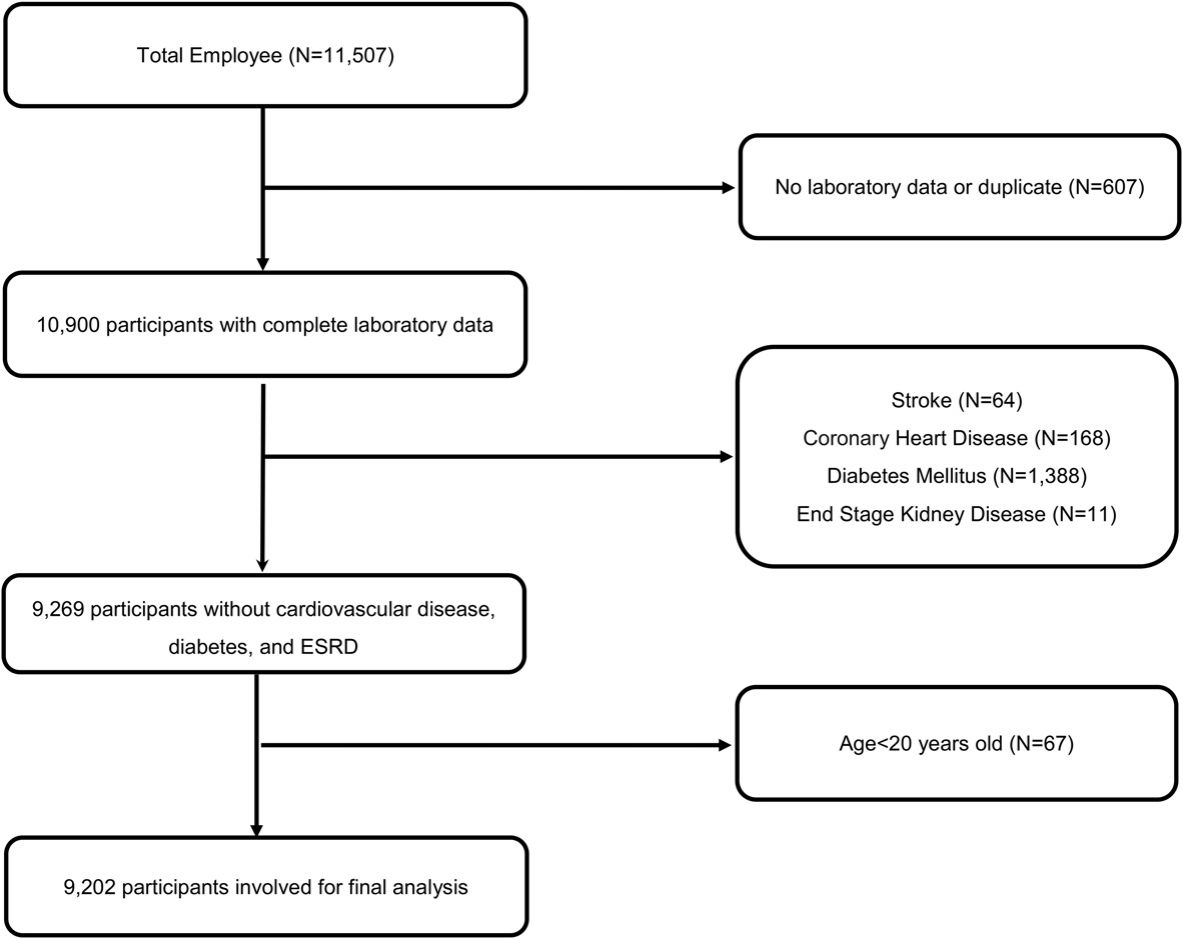
Flow chart of exclusion and inclusion in our study.

In this longitudinal study, spanning from 2014 to 2018, we meticulously collected data from annual health examinations of 11,507 employees, encompassing both medical staff and general personnel, at a major medical center hospital located in Northern Taiwan. Following stringent exclusion criteria that removed participants with cardiovascular diseases, diabetes, and incomplete entries (n=2305) while ensuring privacy through encoding, a total of 9,202 employees aged between 20 and 80 years were included in the analysis. Detailed inclusion and exclusion criteria are shown in **Figure 1**.

### 2.2 General data collection

Vital sign assessments were systematically conducted by trained nursing staff. Waist circumference was measured at the umbilical level in a standing posture to the nearest centimeter using tape with constant tension. Body mass index (BMI) was calculated by dividing the weight of the subjects in kilograms by their height in meters squared (kg/m2) (13). Blood pressure readings, including both systolic blood pressure (SBP) and diastolic blood pressure (DBP), were taken with the participants in a seated position using standard mercury sphygmomanometers following a rest period of five minutes. To ensure accuracy, blood pressure was measured twice per session with a 30- to 60-second interval between measurements, and the average of these readings was recorded (14).

### 2.3 Laboratory measurements

Fasting blood samples were collected after a 12-hour fast in EDTA-containing tubes through venipuncture in a controlled setting. These samples were analyzed to determine the serum levels of total cholesterol, TG, high-density lipoprotein cholesterol (HDL-C), low-density lipoprotein cholesterol (LDL-C), alanine aminotransferase (ALT), and fasting plasma glucose (FBG). TG and TC levels were analyzed using enzymatic methods with a Fuji Dri-Chem analyzer, while HDL-C and LDL-C concentrations were determined using cholesterol assays following dextran sulfate precipitation. FBG was measured using the glucose oxidase method, and ALT levels were assessed through the International Federation of Clinical Chemistry method. The AIP was subsequently calculated using the formula AIP = log10(TG/HDL-C), which is a critical measure for assessing cardiovascular risk by evaluating the balance between triglycerides and HDL cholesterol. Metabolic syndrome is diagnosed based on the International Diabetes Federation Global Consensus Definition, which requires central obesity as a mandatory criterion, defined by WC with ethnicity-specific values, accompanied by any two of the following four factors: elevated TG (≥150 mg/dL or specific treatment for this lipid ab-normality), reduced HDL-C (<40 mg/dL in males and <50 mg/dL in females or specific treatment for this lipid abnormality), elevated blood pressure (SBP ≥130 or DBP ≥85 mm Hg or treatment of previously diagnosed hypertension), and elevated FBG (≥100 mg/dL or previously diagnosed type 2 diabetes) (15, 16).

### 2.4 Statistical analysis

The basic characteristics of categorical variables were expressed as counts and percentages, while those of continuous variables were described using means and standard deviations. AIP levels were categorized into three groups according to tertiles: T1 (≤ 33.3rd percentile), T2 (33.4th to 66.6th percentile), and T3 (> 66.6th percentile), with tertile comparisons conducted using ANOVA for continuous variables and the chi-square test for categorical variables. Pearson’s correlation analysis was utilized to explore the relationship between AIP levels and metabolic syndrome risk factors. Adhering to the STROBE statement, our analysis implemented three models: a univariate logistic regression model (model 1), a model adjusted for age and gender (model 2), and a fully adjusted model incorporating additional adjustments for BMI, SBP, FPG, and LDL-C (model 3). Statistical significance was indicated by two-tailed p-values less than 0.05. All analyses were conducted using PASW SPSS Statistics for Windows, version 26.0 (SPSS Inc., Chicago, IL, USA).

To further justify the statistical methods employed, this study utilized a comprehensive approach to ensure robust and reliable analysis of the relationship between AIP and MetS. The use of ANOVA for continuous variables allowed for detecting significant differences across tertile groups of AIP, while the chi-square test effectively identified associations in categorical data. Pearson’s correlation analysis was chosen to evaluate linear relationships between AIP and individual risk factors for MetS, including BMI, WC, TG, HDL-C, and fasting glucose. Logistic regression models were specifically selected to estimate odds ratios (ORs) for MetS across AIP tertiles, providing a clear understanding of risk magnitudes while adjusting for potential confounders in a stepwise manner. The univariate model (Model 1) identified baseline associations without adjustment, whereas Model 2 accounted for age and gender, addressing demographic variations. Model 3 further incorporated BMI, SBP, FPG, and LDL-C to control for metabolic and cardiovascular confounders, ensuring the robustness of the findings. Gender-specific subgroup analyses were performed to explore potential differences in AIP-MetS associations between men and women, providing critical insights into sex-based variations. The statistical software PASW SPSS Statistics version 26.0 was chosen for its reliability and advanced analytical capabilities, ensuring precise data handling, computation, and result presentation. Its comprehensive suite of statistical tools supported multivariate modeling, subgroup analysis, and hypothesis testing, aligning with the study’s objectives. The use of two-tailed p-values less than 0.05 as the threshold for statistical significance ensured rigorous and conservative interpretations, minimizing the likelihood of Type I errors.

## 3 Results

**Table 1** reveals the basic characteristics of the employees, equally stratified by AIP levels into three tertiles: T1 (<-0.3557), T2 (-0.3557 to -0.0858), and T3 (>-0.0858). The prevalence of MetS and the percentage of men significantly increased with higher AIP levels, with MetS affecting 39.88% of the population in T3 and only 0.81% in T1, and the proportion of men increased to 49.69% in T3 from 9.04% in T1. Continuous variables such as SBP, DBP, BMI, WC, and FPG also demonstrated significant differences across tertiles. Particularly notable were the changes in HDL-C levels, which significantly decreased from 69.88 mg/dL in T1 to 47.02 mg/dL in T3, underscoring the atherogenic risk associated with higher AIP levels.

**Table 1:** Basic characteristics of the employee population at a medical center from 2014 to 2018.

			Atherogenic Index of Plasma						
	Total		T1		T2		T3		*p* value
Case number	9202		3085		3050		3067		
Cutoff value			<-0.3557		-0.3557 to -0.0858		>-0.0858		
Categorical variables									
MetS, N (%)	1408	(15.30%)	25	(0.81%)	160	(5.25%)	1223	(39.88%)	<0.001
Gender, N (%)									<0.001
Men	2592	(28.17%)	279	(9.04%)	789	(25.87%)	1524	(49.69%)	
Women	6610	(71.83%)	2806	(90.96%)	2261	(74.13%)	1543	(50.31%)	
Continuous variables									
	Mean	SD	Mean	SD	Mean	SD	Mean	SD	
Age (years)	41.94	10.13	38.56	9.46	42.45	10.16	44.84	9.76	
SBP (mmHg)	124.66	16.20	118.24	13.68	124.18	15.35	131.58	16.61	<0.001
DBP (mmHg)	73.13	12.28	68.74	10.50	72.41	11.48	78.26	12.81	<0.001
BMI (kg/m^2^)	24.22	4.08	22.07	3.11	23.87	3.57	26.73	4.07	<0.001
WC (cm)	78.53	10.45	72.29	7.68	77.62	8.86	85.71	9.93	<0.001
ALT (U/L)	23.50	19.77	17.07	12.40	21.22	15.86	32.24	25.38	<0.001
FPG (mg/dL)	89.02	18.13	83.77	11.83	87.67	13.13	95.63	24.51	<0.001
HDL-C (mg/dL)	58.57	13.73	69.88	11.42	58.73	9.74	47.02	8.84	<0.001
LDL-C (mg/dL)	115.79	30.43	103.70	25.73	116.76	28.11	126.98	32.46	<0.001
TC (mg/dL)	192.27	33.64	187.55	31.11	190.48	32.10	198.78	36.48	<0.001
TG (mg/dL)	98.00	83.31	49.67	12.07	80.00	16.67	164.52	115.36	<0.001

MetS, metabolic syndrome; SBP, systolic blood pressure; DBP, diastolic blood pressure; BMI, body mass index; WC, waist circumference; FPG, fasting plasma glucose; HDL-C, high-density lipoprotein cholesterol; LDL-C, low-density lipoprotein cholesterol; TC, total cholesterol; TG, triglyceride.

**Table 2** elucidates the correlation between the AIP and MetS risk factors, utilizing Pearson’s coefficient for analysis. Notably, BMI and WC demonstrated strong positive correlations with AIP, with coefficients of 0.48 and 0.55, respectively. Conversely, HDL-C exhibited a strong negative correlation with a coefficient of -0.73, indicating an inverse relationship with the AIP. TG had the highest positive correlation coefficient of 0.77, suggesting a significant link with AIP. These findings highlight the potential of the AIP as a significant marker for assessing metabolic syndrome risk, underscored by its strong associations with key risk factors such as BMI, WC, HDL-C, and TG.

**Table 2:** Correlation between the atherogenic index of plasma and associated risk factors for metabolic syndrome.

Risk factors	Atherogenic Index of Plasma					
	Total		Men		Women	
	Pearson’s coefficient	p value	Pearson’s coefficient	p value	Pearson’s coefficient	p value
Age (year)	0.25	<0.001	0.10	<0.001	0.24	<0.001
SBP (mmHg)	0.34	<0.001	0.18	<0.001	0.30	<0.001
DBP (mmHg)	0.33	<0.001	0.21	<0.001	0.26	<0.001
BMI (kg/m^2^)	0.48	<0.001	0.39	<0.001	0.46	<0.001
WC (cm)	0.55	<0.001	0.41	<0.001	0.47	<0.001
FPG (mg/dL)	0.30	<0.001	0.24	<0.001	0.27	<0.001
HDL-C (mg/dL)	-0.73	<0.001	-0.72	<0.001	-0.68	<0.001
LDL-C (mg/dL)	0.28	<0.001	0.11	<0.001	0.30	<0.001
TC (mg/dL)	0.16	<0.001	0.20	<0.001	0.12	<0.001
TG (mg/dL)	0.77	<0.001	0.73	<0.001	0.87	<0.001

SBP, systolic blood pressure; DBP, diastolic blood pressure; BMI, body mass index; WC, waist circumference; FPG, fasting plasma glucose; HDL-C, high-density lipoprotein cholesterol; LDL-C, low-density lipoprotein cholesterol; TC, total cholesterol; TG, triglyceride.

**Table 3** shows the associations between the AIP and MetS across the three models. According to the unadjusted Model 1, individuals in the second tertile (T2) of the AIP had an odds ratio (OR) of 6.78 with a 95% confidence interval (CI) of 4.43 to 10.36, and those in the third tertile (T3) had an OR of 81.18 with a 95% CI of 54.51 to 121.13, both of which were significant, with p values less than 0.001. When adjusted for age and gender in Model 2, the ORs slightly decreased to 6.28 for T2 and 76.91 for T3, with 95% CIs of 4.10 to 9.62 and 51.31 to 115.28, respectively, maintaining significance at p values less than 0.001. Further adjustments in Model 3 for BMI, SBP, FPG, and LDL resulted in reduced ORs to 3.81 for T2 and 37.14 for T3, with 95% CIs of 2.33 to 6.21 and 23.22 to 59.39, respectively; however, these values were still significant, with p-values less than 0.001. These findings underscore the strong association between higher AIP levels and increased odds of metabolic syndrome, even after adjusting for key demographic and clinical variables.

**Table 3:** The association between atherogenic index of plasma and metabolic syndrome.

		Model 1			Model 2			Model 3	
AIP	OR	95% CI	*p* value	OR	95% CI	*p* value	OR	95% CI	*p* value
T1	1			1			1		
T2	6.78	4.43, 10.36	<0.001	6.28	4.10, 9.62	<0.001	3.81	2.33, 6.21	<0.001
T3	81.18	54.51, 121.13	<0.001	76.91	51.31, 115.28	<0.001	37.14	23.22, 59.39	<0.001

Model 1: unadjusted. Model 2: adjusted for age and gender. Model 3: adjusted for age, gender, BMI, SBP, FPG, and LDL.

AIP, atherogenic index of plasma; OR, odds ratio; CI, confidence interval.

T1, Tertile 1; T2, Tertile 2; T3, Tertile 3.

**Table 4** reveals the gender-specific analysis of the association between the atherogenic index of plasma (AIP) and metabolic syndrome (MetS), showing distinct variations between men and women. For men in the highest AIP tertile (T3), the odds ratio (OR) of developing MetS is significant across all models, decreasing from 42.24 in the unadjusted model to 22.58 in the fully adjusted model. Women, however, exhibit a stronger association, with an OR starting at 94.75 in the unadjusted model and decreasing to 37.18 in the fully adjusted model. The ORs for the second tertile (T2) also show significant differences, where men have lower ORs ranging from 2.53 in the unadjusted model to 1.50 in the fully adjusted model, whereas women’s ORs range from 8.22 to 4.44, indicating a consistently higher risk among women compared to men across all adjustments.

**Table 4:** Gender subgroup analysis of the association between atherogenic index of plasma and metabolic syndrome.

AIP	Model 1			Model 2			Model 3		
	OR	95% CI	*p* value	OR	95% CI	*p* value	OR	95% CI	*p* value
Men									
T1	1.00								
T2	2.53	0.88, 7.28	0.09	2.36	0.82, 6.80	0.11	1.50	0.46, 4.90	0.50
T3	42.24	15.66, 113.96	<0.001	39.48	14.62, 106.62	<0.001	22.58	7.42, 68.68	<0.001
Women									
T1	1.00								
T2	8.22	5.17, 13.08	<0.001	7.34	4.61, 11.68	<0.001	4.44	2.59, 7.61	<0.001
T3	94.75	60.96, 147.28	<0.001	81.62	52.44, 127.05	<0.001	37.18	22.13, 62.45	<0.001

Model 1: unadjusted. Model 2: adjusted for age and gender. Model 3: adjusted for age, gender, BMI, SBP, FPG, and LDL.

AIP, atherogenic index of plasma; OR, odds ratio; CI, confidence interval.

T1, Tertile 1; T2, Tertile 2; T3, Tertile 3.

**Figure 2:**
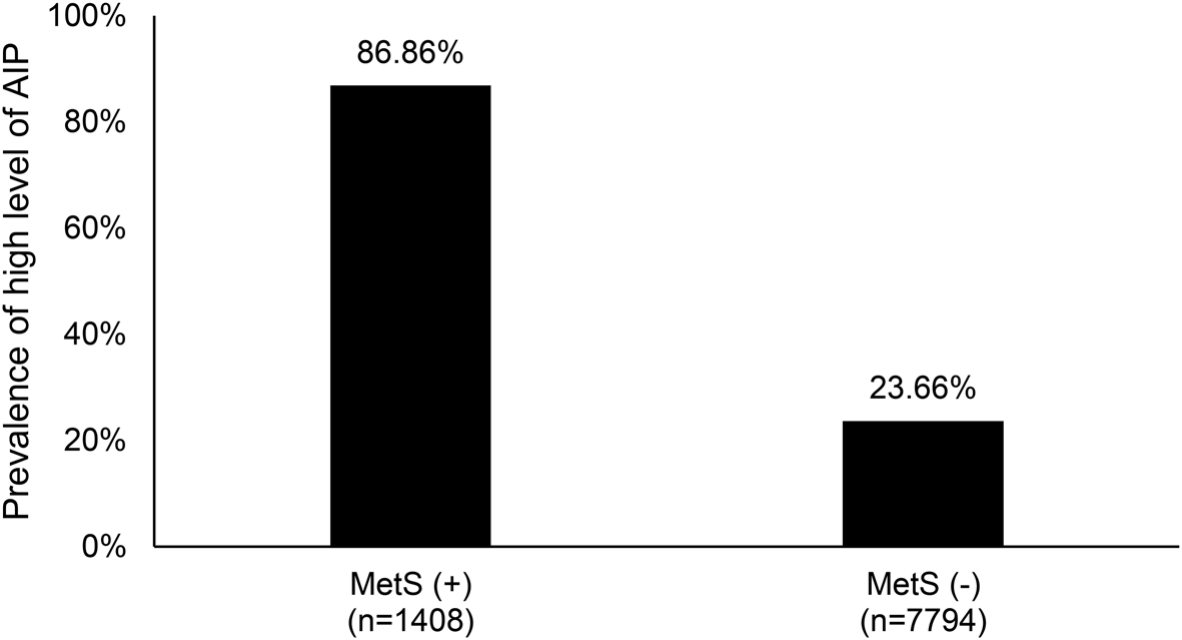
The prevalence of high level (T3) of atherogenic index of plasma between metabolic syn-drome patients and non- metabolic syndrome patients. MetS, metabolic syndrome; AIP, atherogenic index of plasma; T3, Tertile 3.

**Figure 2** illustrates the prevalence of high levels (T3) of the atherogenic index of plasma (AIP) among individuals with and without metabolic syndrome (MetS). The data reveals a significant difference between the two groups. Among the 1,408 subjects diagnosed with metabolic syndrome, 86% exhibited a high AIP level, indicating a strong association between MetS and elevated AIP. In contrast, among the 7,794 sub-jects without metabolic syndrome, only 23% showed high AIP levels, suggesting a lower risk of atherogenic conditions in the absence of MetS. This disparity underscores the link between metabolic syndrome and cardiovascular risk factors.

## 4 Discussion

Our study elucidates the robust association between elevated levels of the AIP and the increased prevalence and risk factors for MetS, demonstrating a graded relationship where higher AIP tertiles significantly correlate with a higher prevalence of MetS and its components, thus reaffirming the potential utility of the AIP as a predictive marker for MetS in a clinical setting. Besides, women show a stronger association between high AIP and MetS risk compared to men, with notably higher odds ratios across all models.

The possible biological mechanisms linking higher AIP to increased MetS risk may involve dyslipidemia, with dyslipidemia’s role in CVD highlighted by the critical diagnostic criteria of triglycerides and HDL-C in MetS and the demonstrated contribution of triglyceride-rich particles to the development and progression of atheromatous plaques (17–19). Triglyceride-rich lipoproteins, such as chylomicrons and very low-density lipoproteins (VLDL), play essential roles in lipid metabolism and energy homeostasis. These lipoproteins transport TG from the intestine and liver to peripheral tissues. The metabolism of these lipoproteins is significantly influenced by enzymes such as lipoprotein lipase, hepatic triglyceride lipase, and endothelial lipase (EL). LPL, anchored to the endothelial surface of capillaries, primarily in adipose tissue and muscle, hydrolyzes the triglycerides in chylomicrons and VLDL into free fatty acids and glycerol, which are then taken up by cells for energy production or storage. This hydrolysis process transforms chylomicrons into chylomicron remnants and VLDL into intermediate-density lipoproteins (IDL) and subsequently into LDL-C. HTGL, mainly found in the liver, further hydrolyzes triglycerides in IDL and chylomicron remnants, facilitating their conversion to LDL-C and their uptake by hepatic receptors, respectively. EL, while primarily hydrolyzing phospholipids, also influences the metabolism of TG-rich lipoproteins by modulating HDL metabolism and indirectly affecting plasma TG levels. Dysfunction in any of these enzymes can lead to elevated plasma triglyceride levels and contribute to metabolic disorders such as hypertriglyceridemia and CVD (20–26).

The observed gender-specific differences in the association between high AIP and MetS risk, as highlighted by notably higher odds ratios in women across all models, merit careful consideration. This variation may stem from inherent biological differences between men and women in lipid metabolism and cardiovascular risk profiles. Typically, women are known to have higher baseline levels of HDL-C, which could modulate the impact of AIP differently compared to men (27–30). Furthermore, hormonal differences, particularly the protective effects of estrogen, may influence lipid and glucose metabolism, altering the risk profile for MetS in premenopausal women (31, 32). However, this protective effect diminishes with age, aligning with an increased MetS risk as seen in the postmenopausal phase (33, 34). Additionally, genetic factors and lifestyle choices, which often vary between genders, might contribute to the observed disparities (35–37).

The strength of our study is its comprehensive approach and nuanced insights into the relationship between AIP and MetS. First, our robust data collection and longitudinal analysis span a significant period, from 2014 to 2018, capturing a large cohort of 9,202 employees with diverse backgrounds, ensuring a broad representation of the population. This extensive dataset allows for a detailed examination of trends and associations over time, enhancing the reliability of our findings. Second, the methodological rigor of categorizing AIP levels into tertiles and conducting a stratified analysis underscores the graded relationship between AIP and MetS, providing a nuanced understanding that higher AIP tertiles significantly correlate with an increased prevalence of MetS and its components. This stratification methodologically enriches the predictive utility of the AIP in a clinical setting. Third, our study benefits from a multivariable adjustment strategy that accounts for various confounding factors, including age, gender, BMI, blood pressure, and FBG levels. This approach ensures that the observed associations between AIP levels and MetS prevalence are not merely artifacts of these confounders but reflect a genuine underlying relationship. Last, the statistical analysis, grounded in Pearson’s correlation and logistic regression models, offers a robust framework for evaluating the strength and significance of the association between AIP and MetS, providing compelling evidence of AIP’s potential as a predictive marker for MetS in diverse clinical settings.

Our study has several inherent limitations. First, relying on a single medical center’s employee cohort might not fully represent the broader population. To mitigate this, we carefully selected a diverse sample of employees, encompassing both medical staff and general personnel, to enhance the generalizability of our findings. Moreover, we applied stringent exclusion criteria to ensure the data’s integrity and reliability, focusing on a well-defined and sizable cohort for analysis. Second, one of the primary constraints is the study’s observational nature, which, despite the robust longitudinal design, may not fully account for all potential confounding variables. To address this issue, we performed a comprehensive statistical analysis, adjusting for a wide range of demographic and clinical factors, such as age, gender, BMI, blood pressure, and FBG levels, to ensure that the observed associations were as accurate as possible. Third, calculating the AIP itself, a recognized marker for cardiovascular risk, depends heavily on accurate triglyceride and HDL cholesterol measurements. Therefore, we ensured that all blood samples were collected and analyzed following stringent, standardized protocols to minimize variability and enhance the reliability of the AIP calculations. Fourth, while our study provides significant insights into the predictive utility of the AIP for MetS, the evolving nature of MetS definitions and criteria poses challenges for longitudinal research. We navigated this by adhering to the most current and widely accepted diagnostic guidelines, allowing for a consistent and relevant assessment of MetS across the study period. Fifth, our study lacks data on dyslipidemia therapies, particularly statins, which could impact the model’s accuracy. Statin use may influence AIP levels and bias the results. According to a study using the Taiwan National Health Insurance Research Database (NHIRD), the use of statins has grown substantially over a decade. In 2011, approximately 6.3% of adults were identified as statin users (38). Sixth, our study highlights AIP’s association with moderate and high-risk MetS cases; future research should include longitudinal designs to evaluate cardiovascular outcomes like myocardial infarction. Additionally, since our study population consists primarily of working adults aged 20 to 65, the lower expected rate of statin use in this group reduces the potential bias on the model’s accuracy (39). Future research should further explore the mechanistic pathways linking AIP and MetS, with a focus on longitudinal and interventional studies to validate the predictive utility of AIP and explore potential therapeutic targets.

In conclusion, our research provides substantial evidence for the significant association between AIP and MetS, emphasizing its predictive value for MetS and related risk factors. The findings reinforce AIP’s utility as a clinically accessible biomarker, facilitating early diagnosis and personalized risk stratification across diverse populations. This study underscores the critical need for longitudinal research to elucidate the mechanisms underlying AIP’s role in metabolic pathways and its temporal relationship with cardiovascular outcomes, such as myocardial infarction. Future studies should incorporate larger, heterogeneous cohorts and advanced imaging techniques to explore AIP’s impact on subclinical atherosclerosis and microvascular complications. Additionally, integrating AIP assessments with genetic, proteomic, and metabolomic profiles could further refine its application in precision medicine. Clinical implications of this work suggest incorporating AIP into routine metabolic health evaluations to enhance early intervention strategies, ultimately improving outcomes in patients at high risk for MetS and cardiovascular diseases. This research marks a pivotal step toward bridging gaps in the literature and advancing metabolic health management frameworks.

## Data Availability

The original contributions presented in the study are included in the article/supplementary material. Further inquiries can be directed to the corresponding author.
